# Catabolic and anabolic periarticular bone changes in patients with rheumatoid arthritis: a computed tomography study on the role of age, disease duration and bone markers

**DOI:** 10.1186/ar4235

**Published:** 2013-05-27

**Authors:** Sophie Aschenberg, Stephanie Finzel, Sarah Schmidt, Sebastian Kraus, Klaus Engelke, Matthias Englbrecht, Jürgen Rech, Georg Schett

**Affiliations:** 1 Department of Internal Medicine 3, University of Erlangen-Nuremberg Ulmenweg 18, 91054 Erlangen, Erlangen, Germany; 2 Institute of Medical Physics, University of Erlangen-Nuremberg, Ulmenweg 18, 91054 Erlangen, Erlangen, Germany

**Keywords:** Rheumatoid arthritis, bone resorption, bone formation, computed tomography, bone biomarkers

## Abstract

**Introduction:**

The aim of this study was to determine the factors, including markers of bone resorption and bone formation, which determine catabolic and anabolic periarticular bone changes in patients with rheumatoid arthritis (RA).

**Methods:**

Forty RA patients received high-resolution peripheral quantitative computed tomography (HR-pQCT) analysis of the metacarpophalangeal joints II and III of the dominantly affected hand at two sequential time points (baseline, one year follow-up). Erosion counts and scores as well as osteophyte counts and scores were recorded. Simultaneously, serum markers of bone resorption (C-terminal telopeptide of type I collagen (CTX I), tartrate-resistant acid phosphatase 5b (TRAP5b)), bone formation (bone alkaline phosphatase (BAP), osteocalcin (OC)) and calcium homeostasis (parathyroid hormone (PTH), 25-hydroxyvitamin D3 (Vit D)) were assessed. Bone biomarkers were correlated to imaging data by partial correlation adjusting for various demographic and disease-specific parameters. Additionally, imaging data were analyzed by mixed linear model regression.

**Results:**

Partial correlation analysis showed that TRAP5b levels correlate significantly with bone erosions, whereas BAP levels correlate with osteophytes at both time points. In the mixed linear model with erosions as the dependent variable, disease duration (*P *<0.001) was the key determinant for these catabolic bone changes. In contrast, BAP (*P *= 0.001) as well as age (*P *= 0.018), but not disease duration (*P *= 0.762), were the main determinants for the anabolic changes (osteophytes) of the periarticular bone in patients with RA.

**Conclusions:**

This study shows that structural bone changes assessed with HR-pQCT are accompanied by alterations in systemic markers of bone resorption and bone formation. Besides, it can be shown that bone erosions in RA patients depend on disease duration, whereas osteophytes are associated with age as well as serum level of BAP. Therefore, these data not only suggest that different variables are involved in formation of bone erosions and osteophytes in RA patients, but also that periarticular bone changes correlate with alterations in systemic markers of bone metabolism, pointing out BAP as an important parameter.

## Introduction

Rheumatoid arthritis (RA) is a chronic inflammatory disease causing bone damage. This process is based on imbalance between bone-resorbing osteoclasts and bone-forming osteoblasts. The imbalance is caused by enhanced expression of inflammatory cytokines, such as tumor necrosis factor (TNF), which foster the differentiation of osteoclasts and hamper formation of osteoblasts. In consequence, repair of bone erosion is limited, with localized deposition of bone at the base of erosions (sclerosis) [1] and growth of small bony spurs [2].

Bone damage in RA is diagnosed and quantified by radiography, allowing semi-quantitative measurement of bone erosion in a reproducible and reliable fashion. Also, the assessment of dynamic changes of erosions is feasible by radiography and represents the basis for demonstrating structure-sparing effects of antirheumatic drugs. It takes, however, several months, until radiographs reveal treatment responses in RA. Thus, it has always been tempting to use markers of bone metabolism to characterize bone changes in RA. Several markers of bone resorption and formation have been assessed in patients with RA, linking them to radiographic data or other outcome variables [3-12]. These data suggested that bone markers might be useful in reflecting bone damage in RA and potentially also in serving as predictors for structural outcome. Still, further work is necessary to understand the association between bone markers and bone structure in RA. In this context, it is surprising that no study has so far linked bone markers to advanced bone imaging.

Herein, we correlated bone markers to high-resolution quantitative computed tomography (HR-pQCT), which we recently developed for characterization of periarticular bone structure in patients with RA [1,2]. This technique is particularly appealing, as it allows simultaneous assessment of catabolic and anabolic bone changes. Also, we analyzed further variables which might be involved in formation of both erosions and osteophytes. We hypothesized that HR-pQCT could allow relating specific bone changes to respective markers of bone resorption and formation in RA patients.

## Methods

### Patients

Forty patients with RA from the Rheumatology Outpatient Clinic of the University Clinic of Erlangen were included. All patients fulfilled the old and American College of Rheumatology (ACR) classification criteria for RA and were assessed at two sequential visits (baseline and after one year). Disease Activity Score (DAS) 28, C-reactive protein (CRP) level and erythrocyte sedimentation rate (ESR) were recorded. The study was performed in accordance with the Declaration of Helsinki. Approvals from the local ethics committee (Ethik-Kommission der Medizinischen Fakultaet der Universitaet Erlangen-Nuremberg) and the national radiation safety agency (Bundesamt für Strahlenschutz) were obtained. All patients gave written informed consent for HR-pQCT measures as well as for blood sample assessments.

### HR-pQCT

All patients received HR-pQCT of the metacarpophalangeal (MCP) joints II and III of the dominantly affected hand at resolution of 82 × 82 × 82 µm voxel size using an XtremeCT scanner (SCANCO Medical AG, Brüttisellen, Switzerland) at baseline and after one year as previously described [1,2]. Scans were assessed for presence and size of bone erosions and osteophytes in overall 530 2D slices. Erosions were defined as a juxta-articular break within the cortical shell, osteophytes as bony protrusions from the juxta-articular cortical shell.

### Imaging data analysis

For image analysis, the palmar, ulnar, dorsal and radial quadrants at both the metacarpal heads as well as the bases of the proximal phalangeal bones II and III were analyzed by two independent readers (SK and KE) unaware of the identity, clinical data and treatment modalities of the patients and also blinded for the sequence of the scans in a total of 16 regions (8 per joint). Numbers of bone erosions and osteophytes in all 16 regions were assessed and semi-quantitatively scored into four grades (0 to 3) as described previously [2]. The results from all 16 compartments were summed up for correlation to bone biomarkers.

### Markers of bone metabolism

Fasting morning blood samples were taken from all patients at the time of imaging. The following bone metabolism parameters were assessed: C-terminal telopeptide of type I collagen (CTX-I), tartrate-resistant acid phosphatase (TRAP) 5b, bone alkaline phosphatase (BAP), 25-hydroxyvitamin D3 (Vit D), osteocalcin (OC) (all measured by MicroVue immunocapture enzyme assay; Quidel Corp., San Diego, CA, USA) and parathyroid hormone (PTH) (by immunoradiometric assay; IBL, Hamburg, Germany).

### Statistics

For data analysis two models were carried out, a partial correlation referring to data at baseline and follow-up and two generalized mixed linear models using delta-values of the two points of time, hence the changes.

The partial correlation analysis was performed between the bone metabolism parameter and the absolute number and score of erosions and osteophytes at baseline and follow-up. We controlled for the influence of sex, age, duration of disease, time interval between baseline and follow-up assessment, ESR, number of swollen joints and number of tender joints as well as antirheumatic treatment.

In order to also control for intrapatient correlation and to model erosion and osteophyte counts at follow-up by changes in bone markers, we set up two generalized mixed linear models with sex, age, disease duration and disease activity (as assessed by the DAS28 at time point 2) as covariates. Changes in bone biomarkers were calculated by subtracting the corresponding parameters at baseline from the parameters at follow-up. Additionally, we included a random intercept and a compound symmetry covariance structure into the model. The corresponding results are expressed as robust regression coefficients, which were favored due to the small sample size and in case of any variable in the model eventually not meeting the methodological requirements. Satterthwaite approximation was used for handling the degrees of freedom. For inter-reader reliability, intraclass correlation coefficient (ICC) was calculated. IBM SPSS Version 19.0 (IBM Inc., Armonk, NY, USA) was used for all statistical analyses; a *P *value of less than 0.05 was considered statistically significant.

## Results

### Demographic characteristics

Mean ± SD age of the participants (75% women) was 55.40 ± 11.77 years, mean disease duration was 4.60 ± 6.42 years. Mean ± SD CRP level at baseline was 7.17 ± 9.46 mg/dl, and mean ± SD ESR level 24.79 ± 15.07 mm/h. Mean DAS28 at baseline was 4.0 ± 1.49 corresponding to a population of moderately active RA patients. A total of 87.5% of the patients received disease-modifying antirheumatic drugs (DMARDs) at baseline (mostly methotrexate (MTX); mean ± SD dose: 15.15 ± 4.42 mg/week), 42.5% were treated with glucocorticoids (mean ± SD dose: 5.07 ± 8.34 mg) and 55% with biologics (TNF blockers: N = 18; tocilizumab N = 4, rituximab N = 2). At follow-up, all patients were treated; 97.5% received DMARDs (mean dose ± SD of MTX: 17.23 ± 4.48 mg/week), 47.5 % glucocorticoids (mean ± SD dose 3.13 ± 3.82 mg) and 62.5 % biologics (TNF blockers: N = 18; tocilizumab N = 5, rituximab N = 3). The time interval between baseline and follow-up scanning was one year (365.80 ± 137.10 days). At one year follow-up, mean DAS28 was 3.65 ± 1.55.

### HR-pQCT analysis

Mean erosion number (sum of erosions detected in the 16 compartments) was 5.70 at baseline with a small and nonsignificant (*P *= 0.338) increase to 6.13 at follow-up. Similarly, erosion scores (the sum of erosion scores in the 16 compartments) showed a tendency to increase after one year with a mean score of 5.30 at baseline and 5.50 at follow-up. Osteophytes were generally small in RA, as reported previously [2], and showed similar dynamics as bone erosions with slight increases at follow-up. Mean osteophyte numbers were 3.40 at baseline and 3.60 at follow-up, mean osteophyte score was 4.18 at baseline and 4.53 at follow-up (Table 1). ICC for inter-reader reliability ranged from 0.57 through 0.95 (worst correlation: the sum of osteophyte counts at baseline; best correlation: the sum of erosion scores at follow-up) with a mean ICC of 0.79.

**Table 1:** Results from the high-resolution peripheral quantitative computed tomography

	Baseline	Follow-up
**Erosion count**	5.70 (± 8.02)	6.13 (± 8.41)

**Erosion score**	5.30 (± 8.02)	5.50 (± 7.66)

**Osteophyte count**	3.40 (± 2.59)	3.60 (± 2.44)

**Osteophyte score**	4.18 (± 3.74)	4.53 (± 3.42)

Mean (± SD) values for erosions and osteophytes at baseline and follow-up.

### Bone biomarkers

For bone resorption, CTX-I and TRAP5b were assessed. Both showed comparable levels at baseline and one year follow-up (CTX-I: baseline 0.34 (± 0.199) ng/ml, follow-up 0.34 (± 0.21) ng/ml; TRAP5b: baseline 2.40 (± 1.25) U/L, follow-up 2.42 (± 1.14) U/L). Bone formation was comparable between baseline and follow-up samples, with only minimal nonsignificant differences for BAP and OC (BAP: baseline 21.92 (± 12.19) U/L, follow-up 20.48 (± 6.91) U/L; OC: at baseline 12.48 (± 7.34) ng/ml, follow-up 11.15 (± 6.02) ng/ml). For calcium metabolism, PTH and vitamin D were analyzed. PTH slightly increased from baseline to follow-up (baseline 14.74 (± 7.98) U/L, follow-up 17.52 (± 9.55) U/L), although differences were not significant, whereas vitamin D showed virtually identical levels at baseline and follow-up (baseline 63.66 (± 29.61) ng/ml, follow-up 63.31 (± 31.14) ng/ml). These data are demonstrated in Table 2.

**Table 2:** Results from the bone biomarker analysis

	Baseline	Follow-up	Normal range
**CTX-I **(ng/ml)	0.34 (± 0.20)	0.34 (± 0.21)	0.14 - 1.35

**TRAP5b **(U/L)	2.40 (± 1.25)	2.42 (± 1.14)	1.50 - 4.30

**BAP **(U/L)	21.92 (± 12.19)	20.48 (± 6.91)	14.20 - 42.70

**OC **(ng/ml)	12.48 (± 7.34)	11.15 (± 6.02)	15.90 - 41.66

**PTH **(pg/ml)	14.74 (± 7.98)	17.52 (± 9.55)	10 - 57

**Vit D **(ng/ml)	63.66 (± 29.61)	63.31 (± 31.14)	20 - 70

Mean (± SD) levels of bone biomarkers at baseline and follow-up. Normal ranges for postmenopausal women are also indicated. CTX-I, C-terminal telopeptide of type I collagen; TRAP5b, tartrate-resistant acid phosphatase 5b; BAP, bone alkaline phosphatase; OC, osteocalcin; PTH, parathyroid hormone; Vit D, 25-hydroxyvitamin D3.

### Correlation between HR-pQCT data and bone metabolism

Partial correlation analysis adjusted for age, sex, disease duration, time interval between baseline and follow-up assessment, ESR, number of swollen and tender joints as well as antirheumatic treatment did reveal significant correlation at the number of bone erosions that correlated to TRAP5b. Correlations were consistent at baseline (r = 0.393*, P *= 0.047) and at follow-up (r = 0.474, *P *= 0.005) (Table 3). Correlations were also found between the severity of erosions and TRAP5b, although statistical significance was only reached at follow-up (r = 0.466, *P *= 0.006). Conversely, osteophyte counts were significantly related to BAP both at baseline (r = 0.446, *P *= 0.022) as well as at follow-up (r = 0.451, *P *= 0.008) (Table 3). Furthermore, correlations between the severity of osteophytes and BAP were observed, albeit they did not reach significance (data not shown). Other bone metabolism parameters did not correlate significantly with structural changes in HR-pQCT.

**Table 3:** Partial correlation of high-resolution peripheral quantitative computed tomography results and bone markers

	Erosion count		Erosion score		Osteophyte count		Osteophyte score	
	
	Baseline	Follow-up	Baseline	Follow-up	Baseline	Follow-up	Baseline	Follow-up
**CTX1**	0.443	0.031	0.319	0.095	0.866	0.981	0.979	0.310

**TRAP5b**	**0.047**	**0.005**	0.109	0.006	0.806	0.766	0.912	0.415

**BAP**	0.218	0.009	0.165	0.010	**0.022**	**0.008**	0.059	0.083

**OC**	0.463	0.029	0.440	0.016	0.620	0.699	0.857	0.884

**PTH**	0.873	0.852	0.833	0.951	0.514	0.014	0.512	0.004

**Vit D**	0.290	0.214	0.408	0.192	0.630	0.397	0.389	0.468

Numbers indicate *P *values; CTX-I, C-terminal telopeptide of type I collagen; TRAP5b, tartrate-resistant acid phosphatase 5b; BAP, bone alkaline phosphatase; OC, osteocalcin; PTH, parathyroid hormone; Vit D, 25-hydroxyvitamin D3.

More stringent analysis with a generalized mixed linear model for erosions showed that only disease duration is positively associated with the number of erosions (*P *<0.001). None of the other independent variables revealed significant associations to the number of erosions in the first model (Table 4). In the second model, we found that changes in BAP are significantly (*P *= 0.001) associated with the number of osteophytes. Moreover, age showed a positive association to the number of osteophytes (*P *= 0.018) (Table 5).

**Table 4:** Mixed linear model regression with erosion counts as dependent variable

Parameter	Regression coefficient	*P *value	95% Confidence interval	
			Lower bound	Upper bound

Intercept	-6.671	0.238	-18.139	4.798

Sex (male)	2.500	0.403	-3.503	8.504

Age (years)	0.104	0.237	-0.075	0.284

Disease duration (years)	**0.868**	**<0.001**	**0.500**	**1.236**

DAS28	0.325	0.556	-0.886	1.535

Change in TRAP5b	-0.215	0.797	-2.118	1.688

Change in BAP	0.035	0.660	-0.186	0.256

Regression coefficients and *P *values are shown for each dependent variable at follow-up. Significant correlations (*P *<0.05) are indicated in bold. DAS28, Disease Activity Score 28; TRAP5b, tartrate-resistant acid phosphatase 5b; BAP, bone alkaline phosphatase.

**Table 5:** Mixed linear model regression with osteophyte counts as dependent variable

Parameter	Regression coefficient	*P *value	95% Confidence interval	
			Lower bound	Upper bound

Intercept	1.090	0.551	-2.646	4.826

Sex (male)	-0.883	0.223	-2.379	0.613

Age (years)	**0.072**	**0.018**	**0.012**	**0.131**

Disease duration (years)	-0.028	0.762	-0.214	0.159

DAS28	-0.390	0.139	-0.913	0.134

Change in TRAP5b	0.091	0.780	-0.593	0.776

Change in BAP	**-0.107**	**0.001**	**-0.178**	**-0.036**

Regression coefficients and *P *values are shown for each dependent variable at follow-up. Significant correlations *(P *<0.05) are indicated in bold. DAS28, Disease Activity Score 28; TRAP5b, tartrate-resistant acid phosphatase 5b; BAP, bone alkaline phosphatase.

## Discussion

No study has yet addressed whether peripheral catabolic and anabolic bone changes in arthritis are related to respective markers of bone turnover. So far, studies were exclusively based on hand radiographs, which are not the most sensitive method for bone analysis. Studies linking arthritic bone changes in HR-pQCT to bone biomarkers have not been undertaken. Moreover, data correlating bone markers to the results from radiographs only addressed catabolic bone changes (erosions) and did not account for anabolic bone changes (osteophytes). In this proof-of-concept investigation, we addressed the relation between catabolic and anabolic bone changes in the HR-pQCT and markers of bone metabolism in arthritis patients on a cross-sectional and longitudinal basis. We showed that catabolic bone changes depend on disease duration but not age, whereas anabolic bone changes depend on age. None of the catabolic bone markers was independently associated with bone erosions, however, BAP, the most widely used marker of bone formation, correlated well with signs of new bone formation.

A number of studies have assessed markers of bone metabolism in patients with RA, some of them relating them to radiographic outcomes. Garnero and colleagues reported an imbalance between bone resorption and bone formation in RA, showing that RA patients developing bone erosions have higher resorption and lower formation markers as compared to patients without erosions [3]. Moreover, resorption markers have shown to predict the risk of progression of structural damage [4-6] and to readily respond to effective anti-inflammatory therapy [7,8]. Importantly, resorption parameters have shown to be related to clinical and laboratory markers of inflammatory activity in RA [9,10], underlining the need for adjustment for these factors when correlating bone biomarkers to structural damage.

Despite TRAP5b being correlated to bone erosions in partial correlation analysis, the mixed linear models did not show resorption markers independently linked to the level of bone erosion, suggesting that they may indeed be influenced by other factors such as systemic rather than local bone loss. Bone erosions, however, were strongly linked to disease duration of RA. This observation supports the concept that the duration of inflammation is critical for peripheral bone loss in RA. In contrast, age and actual disease activity had not much impact on bone erosion.

Local anabolic changes, however, were determined by age and not disease duration. Furthermore, BAP was consistently linked to anabolic changes in the joints. Current data on BAP in RA are conflicting with studies showing decreased levels and such showing no major changes [11-13]. Bone formation is likely to be suppressed in RA [14], which is in accordance with the limited bone responses observed in RA patients assessed by HR-pQCT [1]. Bone formation may not be completely suppressed in RA though, as limited bony proliferations can still be detected, which correlated well with the BAP level in our study. In this context, it is interesting that local production of BAP in RA joints has been reported previously, suggesting that local, albeit poorly effective, BAP production may occur in the arthritic joints [15].

## Conclusions

In summary, these data show that changes in BAP correlate with the anabolic bone changes in RA joints and that disease duration and age also impact the bone microstructure. By using HR-pQCT, it is not only possible to simultaneously depict catabolic and anabolic changes in the joint, but also to correlate these bone changes to serum markers of bone turnover even in rather small patient cohorts. These data are particularly interesting for small proof-of-concept studies aiming to combine strong anti-inflammatory/anti-erosive drug therapy with interventions that foster bone formation and repair.

## Abbreviations

ACR: American College of Rheumatology; BAP: bone alkaline phosphatase; CRP: C-reactive protein; CTX-I: C-terminal telopeptide of type I collagen; DAS: Disease Activity Score; DMARDs: disease-modifying antirheumatic drugs; ESR: erythrocyte sedimentation rate; HR-pQCT: high-resolution peripheral quantitative computed tomography; ICC: intraclass correlation coefficient; MCP: metacarpophalangeal; MTX: methotrexate; OC: osteocalcin; PTH: parathyroid hormone; RA: rheumatoid arthritis; TRAP5b: tartrate-resistant acid phosphatase 5b; TNF: tumor necrosis factor; Vit D: 25-hydroxyvitamin D3.

## Competing interests

The authors declare that they have no competing interests.

## Authors' contributions

SA conducted the study, participated in the data collection, analysis and interpretation and drafted the manuscript. SF participated in the design and conduction of the study, data collection, statistical analysis and interpretation and drafting of the manuscript. SS and SK participated in the data collection and analysis. KE participated in the data analysis. ME participated in the data analysis, data interpretation as well as in the revision of the manuscript content. JR participated in the revision of the final manuscript. GS participated in the study design, data interpretation and the revision of the final manuscript. All authors read and approved the final manuscript.
